# School Performance and Young Adult Crime in a Brazilian Birth Cohort

**DOI:** 10.1007/s40865-022-00214-x

**Published:** 2022-10-11

**Authors:** Rafaela Costa Martins, Helen Gonçalves, Cauane Blumenberg, Bruno Könsgen, Gbènankpon M. Houvèssou, Caroline Carone, Jesus David Gil, Priscila Lautenschläger, Fernando C. Wehrmeister, Ana Maria Baptista Menezes, Joseph Murray

**Affiliations:** 1 Post-Graduate Program in Epidemiology, Federal University of Pelotas, 1160 – 3rd floor, Marechal Deodoro, Brazil; 2 Human Development and Violence Research Centre (DOVE), Federal University of Pelotas, Marechal Deodoro, 1160 – 3 floor, Centro, Pelotas, RS 96020-220 Brazil

**Keywords:** Crime, Academic performance, Violence, Cohort studies

## Abstract

Poor school performance may increase the risk of crime and violence via effects on self-esteem, risky behaviours, peer networks, and perceived stakes in society. Despite very high rates of violence in Latin America, no longitudinal research has addressed this issue in the region. Two aspects of educational performance (grade repetition and school completion) were examined during adolescence in a population-based Brazilian birth cohort study (*n* = 3584). Violent and non-violent crime were measured at age 22 years in confidential self-reports; sociodemographic, family, and individual confounders were measured between birth and age 11 years, and potential mediators were measured at age 18 years. The prevalence of violent and non-violent crimes at 22 years was 8.2% and 3.3%, respectively, referring to acts in the previous twelve months. For youth repeating school grades three times or more, the odds of violent crime were 2.4 (95%CI: 1.6–3.6) times higher than for those who had not repeated any school grade. Youth completing school had a lower risk for both violent (OR = 0.5; 95%CI: 0.4–0.7) and non-violent crime (OR = 0.3; 95%CI: 0.2–0.5), compared to those who did not finish school by the expected age. The protective effect of completing school was independent of the number of grades previously repeated. In conclusion, repeating school grades was associated with increased risk for crime; however, successfully managing to complete school by the expected age was an important protective factor against crime, even after multiple grade repetitions.

**Supplementary Information:**

The online version contains supplementary material available at 10.1007/s40865-022-00214-x.

## Introduction

Violence is a major public health problem worldwide, with the highest rates found in low and middle-income countries in Latin America, the Caribbean, and sub-Saharan Africa (Institute for Health Metrics and Evaluation, 2019; UNODC, 2014). In Latin America, interpersonal violence is the leading cause of premature death among young people, and non-lethal violence carries further major costs to victims, communities, and society, through injuries, consequences for mental health and social and community relations, lost human capital, and financial costs of public and private security (Canudas-Romo & Aburto, 2019; McCollister et al., 2010; Meyer et al., 2014; Perea, 2019; University of Washington & Institute for Health Metrics and Evaluation, 2013; van Dijk et al., 2021). The perpetration of both violent and non-violent crime tends to peak in late adolescence and has been linked to a number of social and health adversities through the life course (Hughes et al., 2020; Murray & Farrington, 2010). The extent to which children and adolescents successfully engage in education is considered a major influence on their life chances, including possible involvement with crime. In this article, we examine the effects of school performance, in terms of grade repetition in adolescence and completion of school, on crime perpetration among young adults in Brazil.

Criminal behaviour can be understood using an ecological model, as it is influenced by broad societal factors, community-level factors, peer, and family environments, as well as individual experiences and characteristics. Some of the most important individual risk factors previously identified in longitudinal studies of crime are impulsiveness and hyperactivity, low intelligence quotient (IQ), low school achievement, poor social skills, positive attitudes towards delinquency, and low resting heart rate (Murray & Farrington, 2010; Murray et al., 2018). Key ways in which educational attainment could influence crime are via school attachment, levels of human capital influencing opportunities for work and non-criminal income, and commitment to non-criminal social roles. In his classic “Social bonding” theory, Travis Hirschi (1969) proposed that crime occurs in the context of disrupted or weakened social bonds (bonds to non-criminal people and social institutions). Hence, poor educational performance during adolescence may reduce youth attachment, commitment, involvement, and belief in the school system (disrupt the youth-school bond) and influence other social relations in a manner that increases propensity towards crime. Also, expectations and aspirations of youth may be negatively affected by experiences of school failure and being held back in school, leading to other risky behaviours, such as substance use, associated with crime. Thus, behaviours may change, future opportunities may become limited, and aspirations curtailed, leading to increased crime perpetration (Costello & Laub, 2020; Hirschi, 1969).

Educational performance may therefore impact later criminal behaviour via multiple indirect pathways suggested by life-course and developmental theories. Considering Sampson and Laub’s age-graded theory of crime (Sampson & Laub, 1993, 2005), poor educational performance during adolescence might represent a biographical turning point with a significant influence on the social bonds hypothesized to determine criminal behaviour. Indeed, their age-graded theory of social control suggests that during childhood and adolescence, school social controls are the primary mechanism limiting delinquent behaviour after family bonds. As such, performing poorly at school may disrupt school attachment, as well as rupture class-peer relationships (if poor performance leads to repeating school grades), and thereby increase the risk of crime. Other life-course criminology theories also hypothesize an important role of educational performance for crime. For example, Farrington’s Integrated Cognitive Antisocial Potential (ICAP) theory (Farrington, 2020) proposes that a long-term “antisocial propensity” develops through childhood and adolescence in relation to a variety of individual, family, and social risk factors, and cites school failure as one of the key influences contributing to this propensity. In line with strain theory, Farrington suggests school failure can push youth towards crime as a means of obtaining material goods and status via illegitimate means—note that this is a different mechanism to the social bonding one specified by Sampson and Laub. In Farrington’s classic longitudinal survey of south London males, the Cambridge Study in Delinquent Development (Farrington, 2003), poor school performance was a strong predictor of crime in adulthood, although mechanisms were not elucidated.

Although the overall association between education and crime is well established, much less is known about potential differences according to child sex and socioeconomic conditions. In a recent Danish registry study on this question, the effects of education were stronger for males than females, and there was some variability by social background (P. Bennett, 2018). In the Dunedin Longitudinal Study, it was also found that the relationship between school involvement and antisocial behaviour was stronger for males than females (T. E. Moffitt et al., 2001). However, this sex difference was not found in a study of risk factors for violence across seven US cities (Peterson & Morgan, 2014), and other studies in the USA suggest there are in fact larger effects of school factors on crime for females (Deschenes & Esbensen, 1999; Saner & Ellickson, 1996), although formal tests of interaction have not always been used. Further tests of possible sex interactions in the effects of school performance on crime are needed in new, large studies.

Several experimental studies have also highlighted the potential for educational investment to reduce delinquency and crime. Perhaps the most famous example concerns even pre-school education. The Perry Preschool Project was a seminal experimental study to report that access to high-quality early education could not only impact on cognition and educational attainment but also reduce crime into adulthood, with major savings in cost–benefit analyses (HighScope Educational Research Foundation, 2021; Schweinhart et al., 1993). Since then, other experimental findings have been consistent (S. Bennett et al., 2018; Lochner & Moretti, 2004; Machin et al., 2011), and a number of longitudinal studies have confirmed associations between low educational performance and conduct disorders and crime (Lynam et al., 1993; Murray & Farrington, 2010; Sabates, 2007; Velez et al., 1989). However, the effects of school achievement on crime have been studied almost exclusively in high-income countries, with relatively low rates of serious violence, and very different social and cultural contexts, compared to regions such as Latin America. Indeed, in a large systematic review conducted in seven languages, there were only two studies on this topic across all low- and middle-income countries, both of which examined final educational attainment as a possible predictor of violence (Murray et al., 2018). The first was conducted in the Philippines and found no association between lower educational attainment and perpetration of intimate partner violence (Fehringer & Hindin, 2009). The second, in South Africa, found a positive association between low educational attainment and family/intimate partner violence, but there was no association with violence against strangers (Thaler & Seekings, 2011). Clearly, much is to be learned about the role of educational performance on crime in the global South.

As well as a lack of data from the global South, life-course mechanisms linking school grade repetition and crime still need specifying, and it is unclear whether effects are similar for males and females, and for children in different family socioeconomic conditions. Finally, an important practical issue is whether achieving adequate school completion by early adulthood can mitigate the effects of earlier poor school performance on crime. Given (i) the major role educational performance might have in influencing crime and violence, (ii) the enormous social and health burden of violence and other crime in Brazil, as in other Latin American countries, and (iii) the lack of longitudinal studies on this topic in this region, we evaluated the association between school performance and youth crime in a large Brazilian birth cohort study, considering several possible mechanisms (problematic alcohol use, drug use, peer drug use, and reduced income in late adolescence), and moderators of the effects, including, sex, family income, and final school achievement.

## Methods

### Study Design

In 1993 a birth cohort study was started in the city of Pelotas, in Southern Brazil. All hospital live births in the urban area of Pelotas (*N* = 5265) were eligible to participate (99% of children were born in hospitals in Pelotas that year). After 16 refusals, 5,249 mothers and children were included in the study. Follow-ups were conducted during childhood and adolescence. The current analyses use data from the perinatal assessment, and follow-ups conducted at ages 4, 11, 18, and 22 years, with retention rates of 87.2%, 87.5%, 81.4%, and 76.3%, respectively. The 4-year assessment was based on a sub-sample of 634 individuals. All other follow-ups included the whole cohort. At 4 and 11 years, participants and their carers (usually mothers) were interviewed at home. At ages 18 and 22, participants were invited to the research clinic for assessments. At each follow-up, trained interviewers applied questionnaires about sociodemographic, economic, health, behavioural, and lifestyle factors. The 22-year-old questionnaire was applied using REDCap (Research Electronic Data Capture) (Harris et al., 2009). Details on the study’s methodology can be found elsewhere (Gonçalves et al., 2014; Victora et al., 2008). This study was approved by the Research Ethics Committee of the Faculty of Medicine at the Federal University of Pelotas (protocol numbers: 029/2003 for 11 years, 05/11 for 18 years, and 1.250.366 for 22 years), and participants and/or parents/guardians received relevant information about the study and signed an informed consent form.

### Measure of Criminal Behaviour

At age 22, participants completed a confidential self-report questionnaire asking about criminal behaviours in the previous 12 months (Murray et al., 2015). This questionnaire was originally developed in Britain, and was previously translated and adapted in Pelotas, Brazil, and validated against official crime records (Murray et al., 2016); it shows expected associations with correlates such as earlier child behaviour problems behaviour (Murray et al., 2015), and low resting heart rate (Murray et al., 2016). As in previous work with this questionnaire, youth were classified as engaging in violent crime if they answered “yes” to having done any of the following acts: stole from person with threat/force, assault, carried a weapon for fights or self-defence, used a weapon, or rape. Youth were classified as having committed a non-violent crime if they answered “yes” to having done at least one of the following acts: stole from person without threat/force, stole from shops/stores, stole from vehicle, stole vehicle, sold drug, sold stolen good, burgled, damaged property, arson.

### Measures of Educational Performance in Adolescence

We examine two aspects of educational performance: grade repetition and school completion. In Brazil, inadequate school performance leads to repeating an academic year until satisfactory performance is attained. At age 11, mothers were asked about how many times their children had repeated a school grade and at age 18 years old the same question was asked to the participants. Using this information, a categorical variable was created indicating the number of school grade repetitions that occurred between 11 and 18 years old (none, once, twice, or three times or more). For females, due to few individuals failing a grade and reporting crimes, the number of school grade repetitions was dichotomized (none or once or more)—which we also presented for males. The impact of grade repetition after age 11 years was studied considering that it may have a larger direct impact on crime than earlier grade repetition, and in order to maintain appropriate temporal ordering of variables in the study (confounders were measured at age 11 years or earlier). School completion is a dichotomous variable that indicates if the participant had finished high school by age 22 (yes or no); in Brazil, adolescents that do not fail any grade typically finish school by age 17 years. A student may continue studying into adulthood to complete all grades. For the current study, in terms of school completion, we measured whether all school grades had been passed up to age 22 years.

### Measures of Confounders

The main confounders measured for this study were all assessed at age 11 years. For neighbourhood conditions, the participant reported whether or not they were afraid of the neighbourhood (yes or no) at age 11. Sociodemographic factors included participant’s family income (in quintiles), maternal schooling in years (0–4, 5–8, 9–11, 12 or more), and skin colour, which is a standard demographic variable measured in Brazil (white, black, brown, or other; in the “other” category there are indigenous and yellow participants). Maternal common mental disorders were assessed using the Self-Reporting Questionnaire (SRQ-20), validated in Brazil (Santos et al., 2010), and a score > 10 was used to identify mothers at elevated risk. Parental physical punishment was measured using a single question responded by participants themselves: “Have you ever been beaten by your parents?”, coded yes or no. Maternal belief in education was assessed by asking the mother: “Until when do you think your child should study?”, and the categories created were up to complete high school, college, post-graduation, or other. Child conduct problems and hyperactivity/inattention problem were measured using the Strengths and Difficulties Questionnaire (SDQ) (Goodman, 2001), previously validated in Brazil (Fleitlich-Bilyk & Goodman, 2004), using > 3 as a cutoff for the conduct problems subscale and ≥ 6 as the cutoff for the hyperactivity/inattention problem subscale. Resting heart rate was measured using a digital monitor (Omron brand, model 711-AC; Beijing, China). Two measures were taken, first at the beginning of the assessment session (after resting for 10 min), the second at the end of the assessment procedures, and the mean of the two measurements was calculated and used in the analyses.

Additional covariates for sensitivity analyses were measured in a sub-sample of children included in the age 4-year psychological assessment. Those variables were IQ (intelligence quotient) and home stimulation. IQ was measured using the WPPSI (Wechsler Preschool and Primary Scale of Intelligence), previously adapted in Argentina (Weschler, 1991) and applied in Brazil (Anselmi et al., 2004). The version used was abbreviated, consisting of two verbal sub-tests (comprehension and arithmetic) and two executive function sub-tests (completing figures and building with blocks). To measure home stimulation and responsive caregiving, the 55-item Early Childhood Home Observation for Measurement of the Environment (EC-HOME) inventory was applied. We summed the two subscales on learning and language to create a final stimulation score for analyses (Bradley, 2015).

### Measures of Potential Mediators

Mediators of effects of school grade repetition were all measured at age 18 years. Hazardous and harmful alcohol use was measured by AUDIT (Alcohol Use Disorders Identification Test) (Saunders et al., 1993), a 10-question-length questionnaire referring to the previous 12 months that was validated in Brazil (Lima et al., 2005). For drug use, participants were asked to complete a confidential questionnaire about the use of any illegal drugs (individually listed) with the following possible answers: (a) never; (b) only tried; (c) not anymore; (d) sometimes; (e) during the weekends; (f) every day or almost every day. Drug use was coded positively at age 18 if any of the following responses were provided: “sometimes”, “during the weekends” or “every day or almost every day”. Friends’ drug use was coded positively if participants responded “yes” about if their friends or colleagues used any illegal drugs (“no” and “don’t know” were combined). Income of the participant was measured by asking “How much money did you earn last month (in Brazilian reais; R$)?”, and was divided into quintiles.

### Statistical Analysis

Analyses were conducted in Stata version 15.1. Results are shown both for the overall sample (main manuscript) and, in supplementary material, we provide results stratified by sex, given some other studies on this topic report results specifically for males or females (Murray et al., 2016; Sweeten et al., 2009). Violent and non-violent crimes were analysed separately. Absolute and relative frequencies of participation in crime, and 95% confidence intervals, were calculated. Chi-squared heterogeneity tests and tests of linear trends were used to calculate *p* values. Logistic regression was used to estimate raw and adjusted odds ratios and their 95% confidence intervals. Models were constructed separately for the effects of grade repetition and school completion, and a further model estimated the effect of school completion adjusting for the number of grades repeated. In sensitivity analyses using data from the 4-year follow-up of the sub-sample (*n* = 632), analyses were weighted given strategic oversampling of low birth weight children—all children with low birth weight were included in the 4-year subsample plus a random sample of children with normal birth weight (Araújo et al., 2010).

Tests of interaction were performed to investigate possible differences in effects of both school performance variables on crime, by participant sex, and by family income at age 11 adopting a logistic model with and without the interaction term using likelihood ratio test. In a further test of interaction, we examined whether the effects of grade repetition on crime differed according to whether or not the participant eventually completed school at expected age. Tests of interaction were conducted using the whole sample (males and females combined).

In mediation analyses, using the *ldecomp* command (Buis, 2010). The total association between grade repetition and crime was decomposed into direct and indirect effects in a logit model. Indirect effects were tested for the following mediators: hazardous and harmful alcohol use, drug use, friends’ drug use, and income.

In sensitivity analyses, we ran the main effects models using Inverse Probability Weighting (IPW), to consider potential bias in the results that might have arisen giving missing data (Online Resource 4 and 5). To derive weights for these analyses, a dummy variable indicating if each individual was included or not in the analyses was used as the outcome in a logistic regression model. Independent variables were inserted in the model using a backward selection approach, excluding those variables with *p* > 0.20, following a three-level hierarchical conceptual model. In the first level, we inserted participant sex, skin colour, and income. In the second level, maternal belief in education of the child, and child hyperactivity, conduct problems, and resting heart were included. In the third level school failure was added. The variables that were associated in multivariable analyses were included in this logistic regression, and weights were derived for the IPW analyses.

## Results

Of the 5249 children included in the birth cohort in 1993, 3810 individuals were followed to age 22 and 3584 (46.5% male; 53.5% female) had complete data on the outcomes for the current analyses. Characteristics of these participants and their families and neighbourhood conditions are shown in Table 1. There were some differences comparing participants included vs. excluded from the analyses, regarding child sex, family income, maternal belief in education, child conduct problems, home stimulation, and the number of grade repetitions, as shown in the Online Resource 1.

**Table 1:** Characteristics of the 1993 Pelotas Birth Cohort Study

	Total sample	
	*N*	% (95%CI)
Afraid of neighbourhood^a^		
No	3004	87.8 (86.9–88.9)
Yes	416	12.2 (11.1–13.3)
Family income (quintiles)^a^		
1st (poorest)	642	18.7 (17.4–20.0)
2nd	717	20.9 (19.5–22.3)
3rd	655	19.1 (17.8–20.4)
4th	694	20.2 (18.9–22.6)
5th (richest)	729	21.2 (19.9–23.6)
Maternal schooling (years)^a^		
0–4	841	24.6 (23.2–26.1)
5–8	1457	42.7 (41.0–44.3)
9–11	775	22.7 (21.3–24.1)
12 or more	343	10.0 (9.1–11.1)
Maternal common mental disorders^a^		
No	2393	70.2 (68.7–71.8)
Yes	1014	29.8 (28.3–31.3)
Maternal belief in education^a^		
Up to complete high school	397	11.6 (10.6–12.7)
College	2218	64.8 (63.1–66.3)
Post-graduation	289	8.4 (7.6–9.4)
Other	521	15.2 (14.1–16.5)
Harsh parenting^a^		
No	1951	57.9 (56.2–59.6)
Yes	1419	42.1 (40.5–43.8)
Child skin colour^a^		
White	2269	66.2 (64.6–67.8)
Black	446	13.0 (11.9–14.2)
Brown	554	16.2 (15.0–17.4)
Other	158	4.6 (4.0–5.4)
Child hyperactivity^a^		
No	3065	91.7 (90.7–92.6)
Yes	278	8.3 (7.4–9.3)
Child conduct problems^a^		
No	2533	75.8 (74.3–77.2)
Yes	809	24.2 (22.8–25.7)
Number of grade repetitions^d^		
0	1088	35.3 (33.7–37.0)
1	663	21.5 (20.1–23.0)
2	721	23.4 (22.0–25.0)
3 or more	607	19.7 (18.4–21.2)
School completion^b^		
Did not finish school	1477	41.3 (39.7–42.9)
Finished school	2102	58.7 (57.1–60.3)
	Mean	SD
Home stimulation (*N* = 472)^c^	36.4	6.7
Child resting heart rate (*N* = 3444)^a^	78.4	10.9
Child IQ (*N* = 464)^c^	93.0	15.2

*CI95%*, 95% confidence interval

^a^Measured at age 11; ^b^measured at age 22; ^c^measured at age 4 years for a sub-sample of the cohort; ^d^variable built with data from 11 to 18 years

Between ages 11 and 18 years, 65.0% of the sample had repeated a school grade at least once (more than 70.0% considering males and almost 60% considering females). By age 22, only 58.4% of youth had completed high school (completed all school grades required in Brazil). The remaining cohort members who had not passed all school grades up to age 22 had not necessarily dropped out of school, but had not passed all school grades up to that age. Of those who did not complete school by 22 years, the mean number of grade repetitions was, as expected, much higher (1.9 times), compared to participants who did complete school (0.9 times; *p* < 0.001).

**Fig. 1:**
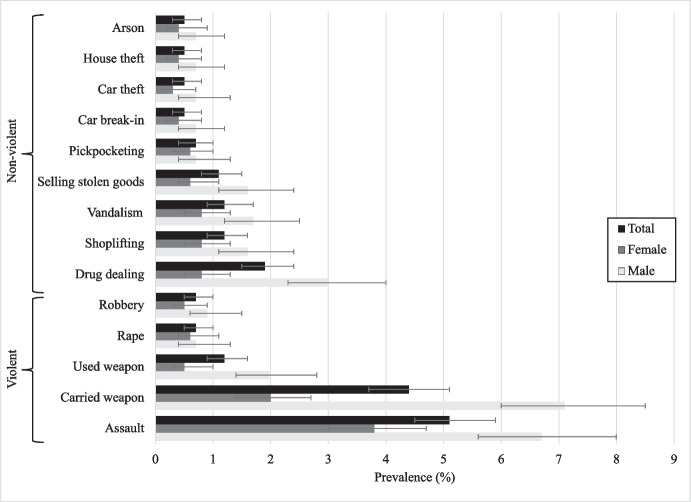
Prevalence of individual violent and non-violent crimes in the 1993 Pelotas Birth Cohort at age 22 years

The prevalence of violent and non-violent crime for the whole sample at 22 years was 8.2% and 3.3%, respectively, referring to acts in the previous twelve months (Table 2). For males, 12.1% (95CI%: 10.6–13.8) reported committing a violent crime, compared to 5.0% (95CI%: 4.0–6.1) of females. For non-violent crime, 4.9% (95CI%: 4.0–5.9) of males and 1.9% (95CI%: 1.4–2.6) of females reported at least one act in the previous 12 months (Online Resource 3). In addition, 3.1% (95CI%: 2.3–4.0) of males and 0.9% (95CI%: 0.6–1.4) of females self-reported both a violent and a non-violent crime (data not shown). Figure 1 shows the individual types of crime reported by the cohort participants. The most common types of crime reported were assault (6.7% for males, 3.8% for females) and carrying a weapon (7.1% for males, 2.0% for females).

**Table 2:** Violent and non-violent crime at 22 years old, according to neighbourhood, family, and childhood characteristics in the 1993 Pelotas Birth Cohort Study

	Total			
	Violent crime		Non-violent crime	
	%	*p*^b^	%	*p*^b^
Afraid of neighbourhood		0.006		0.240
No	7.8 (6.9–8.8)		3.2 (2.6–3.9)	
Yes	12.0 (9.2–15.5)		4.3 (2.7–6.8)	
Family income (quintiles)		0.054		0.016
1st (poorest)	10.9 (8.7–13.6)		5.5 (3.9–7.5)	
2nd	8.0 (6.2–10.2)		3.1 (2.0–4.6)	
3rd	8.7 (6.8–11.1)		3.2 (2.1–4.9)	
4th	6.5 (4.9–8.6)		2.0 (1.2–3.4)	
5th (richest)	7.5 (5.8–9.7)		3.0 (2.0–4.5)	
Maternal schooling (years)		0.630		0.251
0–4	9.3 (7.5–11.4)		4.4 (3.2–6.0)	
5–8	7.8 (6.6–9.3)		3.1 (2.3–4.1)	
9–11	8.5 (6.8–10.7)		2.7 (1.8–4.1)	
12 or more	7.6 (5.2–10.9)		2.9 (1.6–5.3)	
Maternal common mental disorders		0.001		0.011
No	7.2 (6.3–8.3)		2.8 (2.2–3.5)	
Yes	10.8 (9.0–12.8)		4.5 (3.4–6.0)	
Maternal belief in education		0.226		0.024
Up to complete high school	7.6 (5.3–10.6)		3.0 (1.7–5.3)	
College	7.8 (6.8–9.0)		2.8 (2.2–3.6)	
Post-graduation	9.3 (6.5–13.3)		3.5 (1.9–6.3)	
Other	10.4 (8.0–13.3)		5.6 (3.9–7.9)	
Harsh parenting		0.031		0.064
No	7.3 (6.3–8.6)		2.8 (2.2–3.7)	
Yes	9.4 (8.0–11.1)		4.0 (3.1–5.2)	
Child skin colour		0.121		0.019
White	7.8 (6.8–9.0)		2.7 (2.1–3.5)	
Black	11.2 (8.6–14.5)		4.3 (2.7–6.6)	
Brown	7.8 (5.8–10.3)		5.2 (3.7–7.4)	
Other	8.9 (5.3–14.4)		2.5 (1.0–6.6)	
Child hyperactivity		0.207		0.110
No	7.9 (7.0–8.9)		3.1 (2.6–3.8)	
Yes	10.1 (7.0–14.2)		5.0 (3.0–8.3)	
Child conduct problems		< 0.001		0.002
No	7.1 (6.2–8.2)		2.8 (2.2–3.5)	
Yes	11.3 (9.3–13.6)		5.1 (3.8–6.8)	
Home stimulation^a^ (tertiles)		0.048		0.291
1st (least stimulated)	13.6 (9.3–19.6)		6.3 (3.5–11.0)	
2nd	7.5 (4.2–13.1)		4.1 (1.9–8.9)	
3rd (most stimulated)	6.0 (3.2–11.2)		2.7 (1.0–6.9)	
Child resting heart rate (tertiles)		0.906		0.044
1st (lowest)	8.5 (7.0–10.2)		4.4 (3.3–5.7)	
2nd	8.4 (6.9–10.1)		2.6 (1.8–3.7)	
3rd (highest)	8.0 (6.5–9.7)		3.0 (2.1–4.2)	
Child IQ^a^		0.872		0.478
< 90 points	9.6 (6.2–14.6)		5.1 (2.8–9.2)	
≥ 90 points	9.0 (6.1–13.1)		3.4 (1.8–6.4)	
Number of grade repetitions		< 0.001		0.019
0	5.1 (3.9–6.5)		2.0 (1.3–3.1)	
1	7.5 (5.8–9.8)		3.0 (2.0–4.6)	
2	10.1 (8.1–12.6)		3.7 (2.6–5.4)	
3 or more	10.9 (8.6–13.6)		4.6 (3.2–6.6)	
School Completion		< 0.001		< 0.001
Did not finish school	11.7 (10.1–13.4)		5.6 (4.6–6.9)	
Finished school	5.8 (4.9–6.9)		1.7 (1.2–2.4)	
Total	8.2 (7.4–9.2)		3.3 (2.8–4.0)	

*95%CI*, 95% confidence interval

^a^Measured at age 4 years for a sub-sample of the cohort; ^b^Fisher’s exact test

Table 2 shows rates of young adult violent and non-violent crime according to neighbourhood, family, and child characteristics. Violent crimes were more prevalent among participants who were afraid of their neighbourhood, among those whose mothers had common mental disorders at age 11, those who suffered harsh parenting, had had more conduct problems, were least stimulated at age 4, those repeating a school grade 3 times or more, and among those who did not finish school. Non-violent crimes were more common among the poorest individuals, those whose mothers screened positive for common mental disorders at age 11, among people with brown skin colour (followed by black skin colour), those with higher conduct problems, lower resting heart rate, those repeating a school grade 3 times or more, and among those who did not finish school (Table 2). Similar analyses stratified by sex are shown in Online Resource 3.

Table 3 presents regression analyses for the associations between school performance and crime, adjusting for confounders, first for the whole cohort, and then for the sub-sample in which additional confounders were measured at age 4 years (QI variable: *n* = 464, 45.3% male, 54.7% female; home stimulation variable: *n* = 472, 45.1% male, 54.9% female). There was a dose–response relationship between the number of grade repetitions and outcomes of violent and non-violent crimes. For youth repeating school grades three times or more, the odds of violent crime were 2.4 (95%CI: 1.6–3.6) times higher than for those who had not repeated any school grade. Adjusting for additional covariates measured only in the subsample show similar or even larger associations, albeit without statistical significance (Table 3). Online Resources 4, 5, and 6 also show results stratified by sex—using a dichotomous exposure of any grade repetition (repeated at least once versus never repeated a grade) for analyses of female crime, given lower counts of both grade repetitions and crime for females. There was no evidence of a sex difference in the association between grade repetition (comparing “0” versus “1 or more”) and violent crime (*p* = 0.549 in test of interaction) or non-violent crime (*p* = 0.280).

**Table 3:** Associations between school performance and crime for the whole cohort and in a subsample for whom additional covariates were measured at age 4

	Total sample: Model 1		Sub-sample: Model 1		Sub-sample: Model 2	
	Adjusted OR^a^	95%CI	Adjusted OR^a^	95%CI	Adjusted OR^b^	95%CI
Violent crime						
Number grade repetitions	*p* < 0.001^c^		*p* = 0.097^d^		*p* = 0.235^d^	
0	1.0	Ref	1.0	Ref	1.00	Ref
1	1.5	1.0–2.3	1.9	0.9–3.8	2.42	0.69–8.57
2	2.2	1.5–3.3	2.3	1.1–4.7	2.96	0.87–10.09
3 or more	2.4	1.6–3.6	2.0	0.9–4.2	3.16	0.88–11.41
School completion	*p* < 0.001^d^		*p* = 0.007^d^		*p* = 0.265^d^	
Did not finish school	1.0	Ref	1.0	Ref	1.0	Ref
Finished school	0.5	0.4–0.7	0.4	0.2–0.8	0.6	0.2–1.5
Non-violent crime						
Number grade repetitions	*p* = 0.019^c^		*p* = 0.004^c^		*p* = 0.136^d^	
0	1.0	Ref	1.0	Ref	1.0	Ref
1	1.4	0.7–2.7	1.9	0.5–7.3	1.7	0.1–9.2
2	1.8	1.0–2.4	4.8	1.4–17.0	8.8	0.5–22.8
3 or more	2.0	1.1–3.8	5.0	1.4–18.1	5.9	0.7–31.9
School completion	*p* < 0.001^d^		*p* < 0.001^d^		*p* = 0.081^d^	
Did not finish school	1.0	Ref	1.0	Ref	1.0	Ref
Finished school	0.3	0.2–0.5	0.2	0.1–0.5	0.2	0.1–1.3

These analyses include males and females combined.

^a^Adjusted for neighbourhood conditions, family income, maternal schooling, maternal belief in education, maternal mental health problems, harsh parenting, child hyperactivity, child conduct problems, child resting heart rate, child skin colour.

^b^Adjusted for neighbourhood conditions, family income, maternal schooling, maternal belief in education, maternal mental health problems, harsh parenting, child hyperactivity, child conduct problems, child resting heart rate, child skin colour, child IQ at age 4 and home stimulation at age 4.

^c^*p* value for trend.

^d^*p* value for heterogeneity.

**Fig. 2:**
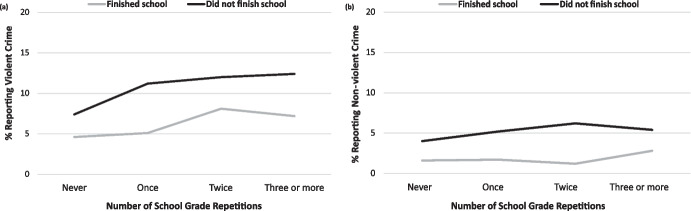
Relationship between school grade repetitions and **a** violent and **b** non-violent crime, stratified by school completion

The association between school completion and crime, adjusted for confounders, is also shown in Table 3. Youth completing school had lower risk for both violent (OR = 0.5; 95%CI: 0.4–0.7) and non-violent crime (OR = 0.3; 95%CI: 0.2–0.5), compared to those who did not finish school by the expected age. As shown in Fig. 2, completing school reduced the risk of participating in crime at every level of grade repetition considered. In other words, school completion was a protective factor against crime regardless of whether adolescents had previously repeated school grades. As one would expect, given the relatively parallel lines in Fig. 2, there was no evidence for an interaction between the number of grade repetitions and completing school in predicting crime (*p* = 0.754 for violent crime, *p* = 0.523 for non-violent crime; males and females combined in the analyses). Results for the association between completing school and crime, stratified by sex, are shown in Online Resource 6, but there was no strong evidence for a sex difference in this association (test of interaction for violent crime *p* = 0.760; non-violent crime *p* = 0.061).

Next, we examined whether the effects of school performance on youth crime differed according to family income. Online Resource 8 shows the relationship between school performance and crime stratified by family income, suggesting that in the wealthiest quintiles (Q3 to Q5), the prevalence of violent crime increased substantially with more grade repetitions, while in the poorest quintiles (Q1 and Q2) the prevalence of violent crime is less variable according to the number of school grade repetitions. This difference was statistically significant (*p* = 0.005 for test of interaction between grade repetition and family income in predicting violent crime), but no difference was observed for non-violent crime (test of interaction, *p* = 0.552). There was also no evidence of interaction between school completion and family income in predicting violent (*p* = 0.645) or non-violent crime (*p* = 0.297).

Lastly, we examined the extent to which the association between school grade repetition and crime was mediated by problematic alcohol use, drug use, friends’ drug use, and income levels for the whole sample (Table 4; for similar analyses for males [for females numbers were too low to estimate stable models], see Online Resource 7). The total effect of grade repetitions between ages 11 and 18 years on the odds of perpetrating crime at age 22, was decomposed into direct effects, and indirect effects via the mediators. These analyses generally showed much larger direct effects and very small indirect effects for each of the mediators (Table 4). The only significant indirect effect observed was for mediation between grade repetition and crime via friends’ drug use. For those repeating a grade three or more times, the estimated indirect effect was OR = 1.1 for violent and OR = 1.2 for non-violent crime, and direct effects were OR = 2.0 and OR = 1.5, respectively.

**Table 4:** Direct and indirect effects of crime and school failure mediated by problematic alcohol use, drug use, friends’ drug use and income

Number of grade repetitions	Violent crime		Non-violent crime	
	Direct	Indirect	Direct	Indirect
	OR (95% CI)	OR (95% CI)	OR (95% CI)	OR (95% CI)
Problematic alcohol use				
0	Ref	Ref	Ref	Ref
1	1.5 (1.0–2.3)	1.1 (1.0–1.1)	1.2 (0.6–2.4)	1.1 (1.0–1.1)
2	2.1 (1.4–3.3)	1.1 (1.0–1.1)	1.6 (0.8–3.2)	1.1 (1.0–1.1)
3 or more	3.0 (1.3–3.1)	1.1 (1.0–1.1)	1.7 (0.8–3.4)	1.1 (1.0–1.2)
Drug use				
0	Ref	Ref	Ref	Ref
1	1.4 (0.9–2.1)	1.0 (1.0–1.1)	1.2 (0.6–2.3)	1.0 (1.0–1.1)
2	2.0 (1.3–2.9)	1.0 (1.0–1.1)	1.5 (0.8–2.8)	1.1 (1.0–1.1)
3 or more	2.0 (1.3–2.9)	1.1 (1.0–1.2)	1.6 (0.8–3.0)	1.1 (1.0–1.3)
Friends’ drug use				
0	Ref	Ref	Ref	Ref
1	1.4 (0.9–2.1)	1.0 (1.0–1.1)	1.2 (0.6–2.3)	1.0 (1.0–1.1)
2	2.0 (1.4–3.0)	1.0 (1.0–1.1)	1.5 (0.8–3.0)	1.0 (0.9–1.1)
3 or more	2.0 (1.3–3.0)	1.1 (1.1–1.2)	1.5 (0.7–3.0)	1.2 (1.1–1.3)
Income level				
0	Ref	Ref	Ref	Ref
1	1.5 (0.8–2.5)	1.0 (1.0–1.1)	0.9 (0.4–2.2)	1.1 (1.0–1.2)
2	1.8 (1.1–3.1)	1.0 (1.0–1.1)	1.4 (0.6–3.1)	1.1 (1.0–1.2)
3 or more	2.2 (1.3–3.9)	1.0 (1.0–1.1)	1.4 (0.6–3.4)	1.1 (1.0–1.3)

## Discussion

The key finding in this population-based, longitudinal study in Brazil is that repeating school grades in adolescence increased the risk for crime in early adulthood, even adjusting for social, demographic, family, and individual confounders and weighting for attrition. Overall, mediation analyses showed that direct effects were larger compared to indirect effects considering substance use variables and youth income. Despite the risks associated with grade repetition, successfully managing to complete school by the expected age, was an important protective factor in reducing the risk for crime, even after multiple grade repetitions. As such, educational support and interventions in schools are potentially important strategies for crime prevention.

Despite improvements in recent years, Brazil is a country with very high levels of both school failure and crime (Numbeo, 2021), and the current study suggests an individual-level link between the two phenomena. The high level of violent compared to non-violent crime found in the current sample at age 22 was also reported at age 18 and was markedly different to a comparable cohort in the UK (using the same instrument) where non-violent crime was more prevalent than violent crime. (Murray et al., 2015) The association between poor academic performance and crime has been documented in several other longitudinal studies in high-income countries (Murray & Farrington, 2010), and the present study confirms this association in Brazil. A major challenge for research on the influence of educational performance and crime is the well-known influence of early child behaviour problems and IQ on crime, as well as various aspects of the family and social environment (Murray & Farrington, 2010). It was notable that, despite extensive adjustment for individual, family, and community variables, poor educational performance carried considerable risk for later violent and non-violent crime in the current study.

The influence of poor educational performance on crime may be explained by a number of different mechanisms, and we examined problematic alcohol use, drug use, friends’ drug use, and income level in late adolescence (age 18) as possible mediators of the effects of grade repetition on crime for males in this study. There was some evidence of indirect effects via friends’ drug use, indicating changes in social networks and association with drugs (strongly tied to criminal groups in Brazil) may be one mechanism by which school failure leads to crime. However, most of the effects of school failure on crime were not explained by this or other mediators in the study, suggesting that other, unmeasured mechanisms were important. Subjective experiences of strain (Agnew, 1992; Sander et al., 2011), social labelling (Farrington & Murray, 2014), and decreased attachment to school are other possible mechanisms that may be important. The main indicator of educational performance we examined was grade repetition, and this reflects individual performance in school examinations each year, but also an institutional response to such performance—i.e. holding back pupils who fail to achieve certain exam criteria. Thus, as well as poor performance possibly affecting crime by its effects on personal psychology (e.g. low self-esteem, decreased attachment to school), this institutional response could also implicate other mechanisms. Specifically, adolescents held back a year then study in a class of younger children, which may rupture bonds with peers who have managed to progress and tie them closer to other adolescents who are also held back. Thus, this educational policy may both challenge adolescents’ self-confidence, and selectively group adolescents who have more difficulties performing well at school closer together. Engaging with older peers involved in antisocial behaviours may be one mechanism by which held-back students attempt to overcome such difficulties, pushing them towards crime. A “maturity gap” between biological age and limited social status in adolescence is exactly the mechanism postulated by Moffitt’s influential theory as leading to emulating older and more antisocial peers during adolescent-onset offending (T. Moffitt, 1993; T. E. Moffitt, 2018). Possibly, the indirect effects of grade repetition on crime via friends’ drug use observed in the current study reflects this process.

Prior studies are mixed regarding whether there are sex differences in the effects of school performance on crime (P. Bennett, 2018; T. E. Moffitt et al., 2001; Peterson & Morgan, 2014; Saner & Ellickson, 1996). For females and males, adolescent development takes place at different speeds, with different biological processes, and in the context of different, gendered social expectations (Feder et al., 2010). Violent gangs in Brazil are primarily comprised by young males (Strocka, 2006), and male socialization that promotes violence is especially evident in macho cultures, as exist in Latin America (Zaitchik & Mosher, 1993; Zdun, 2008). Despite these different biological and social contexts for males and females, the current study did not find evidence that school performance influenced criminal behaviour differently for males and females, which is consistent with one other study of violence and gang membership across seven US cities (Peterson & Morgan, 2014).

The particularly strong influence of grade repetition on violence for adolescents from better-off families was not expected—we hypothesized that education would have more impact on crime in conditions of family adversity. This stronger influence observed for children in better-off families may speak to what Raine has described as the “social push” hypothesis, whereby individual factors (here grade repetition) have the strongest influence on antisocial behaviour in relatively benign social environments (Raine, 2014). By contrast, individual-level factors may be overridden in the context of adversity by stronger social influences.

Despite the concerning association between repeating grades and youth crime documented in this study, a key additional finding was that managing to complete all school years by early adulthood reduced the risk for crime, independently of how many grade repetitions had occurred previously. This demonstrates a protective effect of managing to complete school by the expected age and highlights that even when adolescents are failing grades multiple times, continued educational support can make a vital difference. It speaks to the final outcome of schooling as highly important in terms of an educational qualification that can support youth in the world of employment and non-criminal social activities. Notably, a large proportion (some 40%) of youth in this cohort did not manage to complete high school by young adulthood.

Four types of policies seem particularly important to support adolescents struggling in school and prevent subsequent crime. First, providing high-quality education for all children from an early age can minimize the number of youths who struggle to meet key performance criteria. This can start in preschool, when supportive interventions can have major cost–benefit returns over the life course. Early investment in literacy and numeracy are considered foundational skills on which most other educational outcomes depend, and in contexts of high inequality, like Brazil, it is particularly important such skills are supported among all children in this critical early period (Crouch, 2020). However, reducing the risk of educational failure is critical throughout childhood and into adolescence, and a number of extra-curricular interventions are possible to support child learning in this regard. For example, an after-school programme focused on academic and recreational activities for 9-year-old children in the USA found significant benefits in terms of academic skills and social adjustment (Posner & Vandell, 1994). A second issue for policy is to address other causes of difficulties for students, such as mental health and behavioural problems that affect their capacity to engage in school learning need to be addressed by specialized professionals. Schools need to work closely with families and other public services to address student difficulties that may arise from family or community problems. Third, although the policy of grade retention itself could not be isolated as the mechanism in this study, this policy, which widens the gap between age and social roles, needs further careful evaluation in terms of potential costs to social development (and potential future crime), as well as educational issues. A recent initiative in Pelotas city where the current study was conducted, known as “Construindo Saberes” (Constructing Knowledge), aims to minimize the problem of age/grade distortion among elementary and middle school students, offering extra Portuguese and math lessons to improve school performance and, subsequently, school dropout (Borges et al., 2020). Forth, for students who do fail school examinations, even repeatedly, considerable support must be invested to maximize their continued engagement in and completion of school—completion by early adulthood clearly has major benefits (indicated by lower levels of crime) no matter the level of performance-related difficulties through adolescence.

The strengths of this study include first the design of a population-based birth cohort; second a 22-year follow-up with a relatively high rate of retention; and third, adjustment for important potential confounders. Furthermore, the majority of longitudinal studies of crime have included only males, and nearly all have been conducted in high-income countries; we present data for both sexes in a different setting with high rates of violence. The consideration of both performance during school years, as well as final attainment, is also important. Limitations of the study are as follows. Comparing participants in the analytic sample to those excluded because of death, attrition, or missing data on key variables, those included were generally more socially advantaged than excluded participants; this could mean estimates of exposure and outcome are underestimated and associations could be misspecified. School failure and crime outcomes were self-reported, which could introduce recall bias, social desirability bias, or shared reporter bias. IQ and some other early life variables could only be considered in a small sub-sample for which results were non-significant. Ideally, criminal records would also be used, but those were not available at the time of the analyses.

In conclusion, school performance is an important influence on youth crime. The role of the education sector is clearly crucial in reducing crime and violence. Educators can play a major role by supporting school progress and working with families, health, and other public sectors to support children and adolescents both preventively, and once difficulties with progress have been identified. Even after students show multiple years of educational difficulty, school completion is a major landmark that students, families, teachers, and welfare support systems should aim for.

Online Resource 1 Comparison of analytic sample (n = 3,584) and participants excluded from the analyses (n = 1,665).

Online Resource 2 Characteristics of the 1993 Pelotas Birth Cohort Study stratified by sex.

Online Resource 3 Violent and non-violent crime at 22 years old, according to neighbourhood, family, and childhood characteristics in the 1993 Pelotas Birth Cohort Study stratified by sex.

Online Resource 4. Crude (n = 1,426 for grade repetitions and 1,662 for school completion) and adjusted (n = 1,352) associations between educational performance and crime among males in the 1993 Pelotas Birth Cohort Study.

Online Resource 5. Crude (n = 1,620 for grade repetitions and 1,666 for school completion) and adjusted (n = 1,581) associations between educational performance and crime among females in the 1993 Pelotas Birth Cohort Study.

Online Resource 6. Overall crude (n = 3,079 for grade repetitions and n = 3,579 for school completion) and adjusted (n = 2,933 for grade repetitions and n = 2,929 for school completion) associations between school performance and crime and stratified by males (crude: n = 1,426 for grade repetitions and 1,662 for school completion; adjusted: n = 1,352) and females (crude: n = 1,653 for grade repetitions and n = 1,917 for school completion; adjusted n = 1,581) in the 1993 Pelotas Birth Cohort Study.

Online Resource 7. Direct and indirect effects of crime and school failure mediated by problematic alcohol use, drug use, friends’ drug use and income in males.

Online Resource 8. Relationship between (a) school grade repetitions and violent crime; (b) school grade repetition and non-violent crime; (c) school completion and violent crime; (d) school completion and non-violent crime by family income.

## Supplementary Information

Below is the link to the electronic supplementary material.

Supplementary file1 (DOCX 21 KB)

Supplementary file2 (DOCX 21 KB)

Supplementary file3 (DOCX 25 KB)

Supplementary file4 (DOCX 21 KB)

Supplementary file5 (DOCX 20 KB)

Supplementary file6 (DOCX 18 KB)

Supplementary file7 (DOCX 16 KB)

Supplementary file8 (PNG 502 KB)
