# Supplementary material for: School Performance and Young Adult Crime in a Brazilian Birth Cohort
Source: J Dev Life Course Criminol. 2022 Oct 11;8(4):647–68. doi: 10.1007/s40865-022-00214-x (PMC9825356; doi:10.1007/s40865-022-00214-x)
Supplement: Supplementary file 1 — Supplementary file1 (DOCX 21 KB) [file 40865_2022_214_MOESM1_ESM.docx]

Article title: School performance and young adult crime in a Brazilian birth cohort
Journal name: Journal of Development and Life-course Criminology
Author names: [*hidden due to blindness*]
Affiliation: [*hidden due to blindness*]
E-mail address of the corresponding author: [*hidden due to blindness*]

**Supplementary Table 1.** Comparison of analytic sample (n = 3,584) and participants excluded from the analyses (n = 1,665)

|  | Participants included in the analyses | | Excluded participants | |  |
| --- | --- | --- | --- | --- | --- |
|  | N | % | N | % | *p*^a^ |
| Child sex |  |  |  |  | <0.001 |
| Male | 1667 | 46.5 (44.9 – 48.2) | 936 | 56.3 (53.9 – 58.6) |  |
| Female | 1917 | 53.5 (51.9 – 55.1) | 728 | 43.8 (41.4 – 46.2) |  |
| Child skin colour |  |  |  |  | 0.240 |
| White | 2269 | 66.2 (64.6 – 67.8) | 684 | 68.9 (65.9 – 71.7) |  |
| Black | 446 | 13.0 (11.9 – 14.2) | 109 | 11.0 (9.2 – 13.1) |  |
| Brown | 554 | 16.2 (15.0 – 17.4) | 150 | 15.1 (13.0 – 17.5) |  |
| Other | 158 | 4.6 (4.0 – 5.4) | 50 | 5.0 (3.8 – 6.6) |  |
| Family income (quintiles) |  |  |  |  | <0.001 |
| 1st (poorest) | 642 | 18.7 (17.4 – 20.0) | 255 | 25.1 (22.6 – 27.9) |  |
| 2nd | 717 | 20.9 (19.5 – 22.3) | 205 | 20.2 (17.8 – 22.8) |  |
| 3rd | 655 | 19.1 (17.8 – 20.4) | 204 | 20.1 (17.8 – 22.7) |  |
| 4th | 694 | 20.2 (18.9 – 21.6) | 190 | 18.7 (16.4 – 21.2) |  |
| 5th (richest) | 729 | 21.2 (19.9 – 22.6) | 161 | 15.9 (13.7 – 18.2) |  |
| Afraid of neighbourhood |  |  |  |  | 0.841 |
| No | 3004 | 87.8 (86.7 – 88.9) | 876 | 87.6 (85.4 – 89.5) |  |
| Yes | 416 | 12.2 (11.1 – 13.3) | 124 | 12.4 (10.5 – 14.6) |  |
| Maternal belief in education |  |  |  |  | <0.001 |
| Up to complete high school | 397 | 11.6 (10.6 – 12.7) | 159 | 15.9 (13.8 – 18.3) |  |
| College | 2218 | 64.8 (63.1 – 66.3) | 566 | 56.5 (53.5 – 59.6) |  |
| Post-graduation | 289 | 8.4 (7.6 – 9.4) | 75 | 7.5 (6.0 – 9.3) |  |
| Other | 521 | 15.2 (14.1 – 16.5) | 201 | 20.1 (17.7 – 22.7) |  |
| Child hyperactivity |  |  |  |  | 0.400 |
| No | 3065 | 91.7 (90.7 – 92.6) | 871 | 90.8 (88.8 – 92.5) |  |
| Yes | 278 | 8.3 (7.4 – 9.3) | 88 | 9.2 (7.5 – 11.2) |  |
| Child conduct problems |  |  |  |  | 0.033 |
| No | 2533 | 75.8 (74.3 – 77.2) | 693 | 72.4 (69.5 – 75.2) |  |
| Yes | 809 | 24.2 (22.8 – 25.7) | 264 | 27.6 (24.9 – 30.5) |  |
| Home stimulation^b^ (tertiles) |  |  |  |  | 0.008 |
| 1^st^ (least stimulated) | 151 | 32.0 (27.9 – 36.4) | 72 | 45.0 (37.5 – 52.8) |  |
| 2^nd^ | 171 | 36.2 (32.0 – 40.7) | 42 | 26.3 (20.0 – 33.6) |  |
| 3^rd^ (most stimulated) | 150 | 31.8 (27.7 – 36.1) | 46 | 28.8 (22.3 – 36.3) |  |
| Child resting heart rate (tertiles) |  |  |  |  | 0.946 |
| 1^st^ (lowest) | 1195 | 34.8 (33.2 – 36.4) | 349 | 34.8 (31.9 – 37.8) |  |
| 2^nd^ | 1135 | 33.1 (31.5 – 34.7) | 336 | 33.5 (30.7 – 36.5) |  |
| 3^rd^ (highest) | 1103 | 32.1 (30.6 – 33.7) | 317 | 31.6 (28.8 – 34.6) |  |
| Child IQ^b^ |  |  |  |  | 0.068 |
| <90 points | 197 | 42.5 (38.0 – 47.0) | 76 | 51.0 (43.0 – 59.0) |  |
| ≥90 points | 267 | 57.5 (53.0 – 62.0) | 73 | 49.0 (41.1 – 57.0) |  |
| Number grade repetitions |  |  |  |  | 0.007 |
| 0 | 1125 | 35.3 (33.7 – 37.0) | 88 | 29.2 (24.4 – 34.6) |  |
| 1 | 682 | 21.4 (20.0 – 22.9) | 87 | 28.9 (24.1 – 34.3) |  |
| 2 | 747 | 23.5 (22.0 – 25.0) | 77 | 25.6 (21.0 – 30.8) |  |
| 3 or more | 632 | 19.8 (18.5 – 21.3) | 49 | 16.3 (12.5 – 20.9) |  |

^a^chi-squared test; ^b^sub-sample individuals

Note: We did not compare variables with small sample size in at least one 2x2 cell (<10 individuals) between analyzed and excluded participants (maternal schooling, maternal common mental disorders and harsh parenting).
