# Supplementary material for: School Performance and Young Adult Crime in a Brazilian Birth Cohort
Source: J Dev Life Course Criminol. 2022 Oct 11;8(4):647–68. doi: 10.1007/s40865-022-00214-x (PMC9825356; doi:10.1007/s40865-022-00214-x)
Supplement: Supplementary file 2 — Supplementary file2 (DOCX 21 KB) [file 40865_2022_214_MOESM2_ESM.docx]

Article title: School performance and young adult crime in a Brazilian birth cohort
Journal name: Journal of Development and Life-course Criminology
Author names: [*hidden due to blindness*]
Affiliation: [*hidden due to blindness*]
E-mail address of the corresponding author: [*hidden due to blindness*]

**Supplementary Table 2.** Characteristics of the 1993 Pelotas Birth Cohort Study stratified by sex

|  | MALES | | FEMALES | | |
| --- | --- | --- | --- | --- | --- |
|  | N | % (95%CI) | N | % (95%CI) |  |
| Afraid of neighbourhood^a^ |  |  |  |  |  |
| No | 1386 | 87.3 (85.6 – 88.9) | 1618 | 88.3 (86.7 – 89.7) |  |
| Yes | 201 | 12.7 (1.11 – 14.4) | 215 | 11.7 (10.3 – 13.3) |  |
| Family income (quintiles)^a^ |  |  |  |  |  |
| 1st (poorest) | 292 | 18.3 (16.5 – 20.3) | 350 | 19.0 (17.3 – 20.9) |  |
| 2nd | 330 | 20.7 (18.8 – 22.8) | 387 | 21.0 (19.2 – 22.9) |  |
| 3rd | 297 | 18.6 (16.8 – 20.6) | 358 | 19.4 (17.7 – 21.3) |  |
| 4th | 321 | 20.1 (18.2 – 22.2) | 373 | 20.3 (18.5 – 22.2) |  |
| 5th (richest) | 355 | 22.3 (20.3 – 24.4) | 374 | 20.3 (18.5 – 22.2) |  |
| Maternal schooling (years)^a^ |  |  |  |  |  |
| 0-4 | 381 | 24.0 (22.0 – 26.2) | 460 | 25.1 (23.2 – 27.2) |  |
| 5-8 | 667 | 42.1 (39.7 – 44.5) | 790 | 43.2 (40.9 – 45.4) |  |
| 9-11 | 374 | 23.6 (21.6 – 25.8) | 401 | 21.9 (20.1 – 23.9) |  |
| 12 or more | 163 | 10.3 (8.9 – 11.9) | 180 | 9.8 (8.6 – 11.3) |  |
| Maternal common mental disorders^a,†^ |  |  |  |  |  |
| No | 1015 | 70.0 (67.7 – 72.2) | 1288 | 70.5 (68.3 – 72.5) |  |
| Yes | 474 | 30.0 (27.8 – 32.3) | 540 | 29.5 (27.5 – 31.7) |  |
| Maternal belief in education^a^ |  |  |  |  |  |
| Up to complete high school | 206 | 13.0 (11.4 – 14.7) | 191 | 10.4 (9.1 – 11.9) |  |
| College | 1026 | 64.5 (62.1 – 66.9) | 1192 | 65.0 (62.8 – 67.1) |  |
| Post-graduation | 122 | 7.7 (6.5 – 9.1) | 167 | 9.1 (7.9 – 10.5) |  |
| Other | 236 | 14.8 (13.2 – 16.7) | 285 | 15.5 (13.9 – 17.3) |  |
| Harsh parenting^a^ |  |  |  |  |  |
| No | 874 | 55.8 (53.4 – 58.3) | 1077 | 59.7 (57.4 – 61.9) |  |
| Yes | 691 | 44.2 (41.7 – 46.6) | 728 | 40.3 (38.1 – 42.6) |  |
| Child skin colour^a^ |  |  |  |  |  |
| White | 1087 | 68.3 (66.0 – 70.6) | 1182 | 64.4 (62.2 – 66.5) |  |
| Black | 199 | 12.5 (11.0 – 14.2) | 247 | 13.5 (12.0 – 15.1) |  |
| Brown | 235 | 14.8 (13.1 – 16.6) | 319 | 17.4 (15.7 – 19.2) |  |
| Other | 70 | 4.4 (3.5 – 5.5) | 88 | 4.8 (3.9 – 5.9) |  |
| Child hyperactivity^a^ |  |  |  |  |  |
| No | 1384 | 90.0 (88.4 – 91.4) | 1681 | 93.1 (91.9 – 94.2) |  |
| Yes | 154 | 10.0 (8.6 – 11.6) | 124 | 6.9 (5.8 – 8.1) |  |
| Child conduct problems^a^ |  |  |  |  |  |
| No | 1114 | 72.5 (70.2 – 74.7) | 1419 | 78.6 (76.7 – 80.5) |  |
| Yes | 423 | 27.5 (25.3 – 29.8) | 386 | 21.4 (19.6 – 23.3) |  |
| Number of grade repetitions^d^ |  |  |  |  |  |
| 0 | 396 | 27.8 (25.5 – 30.2) | 692 | 41.9 (39.5 – 44.3) |  |
| 1 | 290 | 20.3 (18.3 – 22.5) | 373 | 22.6 (20.6 – 24.7) |  |
| 2 | 368 | 25.8 (23.6 – 28.1) | 353 | 21.4 (19.5 – 23.4) |  |
| 3 or more | 372 | 26.1 (23.9 – 28.4) | 235 | 14.2 (12.6 – 16.0) |  |
| School completion^b^ |  |  |  |  |  |
| Did not finish school | 800 | 48.1 (45.7 – 50.5) | 677 | 35.3 (33.2 – 37.5) |  |
| Finished school | 862 | 51.9 (49.5 – 54.3) | 1240 | 64.7 (62.5 – 66.8) |  |
|  | **Mean** | **SD** | **Mean** | **SD** |  |
| Home stimulation (N=472)^c^ | 35.9 | 6.7 | 36.8 | 6.6 |  |
| Child resting heart rate (N=3444)^a^ | 76.3 | 10.7 | 80.2 | 10.9 |  |
| Child IQ (N=464)^c^ | 91.5 | 14.9 | 94.2 | 15.4 |  |

Notes: CI95% = 95% Confidence Interval

^a^measured at age 11; ^b^measured at age 22; ^c^measured at age 4 years for a sub-sample of the cohort; ^d^variable built with data from 11 to 18 years
