# Supplementary material for: School Performance and Young Adult Crime in a Brazilian Birth Cohort
Source: J Dev Life Course Criminol. 2022 Oct 11;8(4):647–68. doi: 10.1007/s40865-022-00214-x (PMC9825356; doi:10.1007/s40865-022-00214-x)
Supplement: Supplementary file 3 — Supplementary file3 (DOCX 25 KB) [file 40865_2022_214_MOESM3_ESM.docx]

Article title: School performance and young adult crime in a Brazilian birth cohort
Journal name: Journal of Development and Life-course Criminology
Author names: [*hidden due to blindness*]
Affiliation: [*hidden due to blindness*]
E-mail address of the corresponding author: [*hidden due to blindness*]

**Supplementary Table 3.** Violent and non-violent crime at 22 years old, according to neighbourhood, family, and childhood characteristics in the 1993 Pelotas Birth Cohort Study stratified by sex

|  | MALES | | | | | FEMALES | | | | |
| --- | --- | --- | --- | --- | --- | --- | --- | --- | --- | --- |
|  | Violent crime | | Non-violent crime | | | Violent crime | | | Non-violent crime | |
|  | % | *p*^b^ | % | *p*^b^ | % | | *p*^b^ | % | | *p*^b^ |
| Afraid of neighbourhood |  | 0.021 |  | 0.483 |  | | 0.133 |  | | 0.290 |
| No | 11.5 (9.9 – 13.3) |  | 4.8 (3.8 – 6.0) |  | 4.6 (3.7 – 5.8) | |  | 1.8 (1.3 – 2.6) | |  |
| Yes | 17.4 (12.8 – 23.3) |  | 6.0 (3.4 – 10.2) |  | 7.0 (4.3 – 11.3) | |  | 2.8 (1.3 – 6.1) | |  |
| Family income (quintiles) |  | 0.426 |  | 0.184 |  | | 0.002 |  | | 0.066 |
| 1st (poorest) | 13.0 (9.6 – 17.4) |  | 7.5 (5.0 – 11.2) |  | 9.1 (6.5 – 12.7) | |  | 3.7 (2.2 – 6.3) | |  |
| 2nd | 11.2 (8.2 – 15.1) |  | 5.2 (3.2 – 8.1) |  | 5.2 (3.4 – 7.9) | |  | 1.3 (0.5 – 3.1) | |  |
| 3rd | 14.8 (11.2 – 19.3) |  | 5.1 (3.1 – 8.2) |  | 3.6 (2.1 – 6.2) | |  | 1.7 (0.8 – 3.7) | |  |
| 4th | 10.0 (7.1 – 13.8) |  | 3.4 (1.9 – 6.1) |  | 3.5 (2.0 – 5.9) | |  | 0.8 (0.3 – 2.5) | |  |
| 5th (richest) | 12.1 (9.1 – 15.9) |  | 3.9 (2.4 – 6.6) |  | 3.2 (1.8 – 5.6) | |  | 2.1 (1.1 – 4.2) | |  |
| Maternal schooling (years) |  | 0.920 |  | 0.271 |  | | 0.098 |  | | 0.754 |
| 0-4 | 11.8 (8.9 – 15.5) |  | 6.8 (4.7 – 9.8) |  | 7.2 (5.1 – 9.9) | |  | 2.4 (1.3 – 4.3) | |  |
| 5-8 | 12.3 (10.0 – 15.0) |  | 4.7 (3.3 – 6.5) |  | 4.1 (2.9 – 5.7) | |  | 1.8 (1.1 – 3.0) | |  |
| 9-11 | 13.1 (10.0 – 16.9) |  | 4.0 (2.4 – 6.6) |  | 4.2 (2.7 – 6.7) | |  | 1.5 (0.7 – 3.3) | |  |
| 12 or more | 11.0 (7.1 – 16.9) |  | 3.7 (1.7 – 8.0) |  | 4.4 (2.2 – 8.6) | |  | 2.2 (0.8 – 5.8) | |  |
| Maternal common mental disorders |  | 0.007 |  | 0.007 |  | | 0.057 |  | | 0.575 |
| No | 10.7 (9.0 – 12.6) |  | 3.9 (2.9 – 5.2) |  | 4.3 (3.3 – 5.5) | |  | 1.8 (1.2 – 2.7) | |  |
| Yes | 15.6 (12.6 – 19.2) |  | 7.2 (5.2 – 9.9) |  | 6.5 (4.7 – 8.9) | |  | 2.2 (1.3 – 3.9) | |  |
| Maternal belief in education |  | 0.291 |  | 0.051 |  | | 0.450 |  | | 0.425 |
| Up to complete high school | 11.7 (7.9 – 16.8) |  | 4.4 (2.3 – 8.2) |  | 3.1 (1.4 – 6.8) | |  | 1.6 (0.5 – 4.8) | |  |
| College | 11.3 (9.5 – 13.4) |  | 4.1 (3.0 – 5.5) |  | 4.8 (3.7 – 6.2) | |  | 1.7 (1.1 – 2.6) | |  |
| Post-graduation | 14.8 (9.5 – 22.2) |  | 5.7 (2.8 – 11.6) |  | 5.4 (2.8 – 10.0) | |  | 1.8 (0.6 – 5.4) | |  |
| Other | 15.3 (11.2 – 20.4) |  | 8.5 (5.5 – 12.8) |  | 6.3 (4.0 – 9.8) | |  | 3.2 (1.7 – 6.0) | |  |
| Harsh parenting |  | 0.161 |  | 0.160 |  | | 0.218 |  | | 0.385 |
| No | 11.1 (9.2 – 13.4) |  | 4.2 (3.1 – 5.8) |  | 4.3 (3.2 – 5.7) | |  | 1.7 (1.1 – 2.6) | |  |
| Yes | 13.5 (11.1 – 16.2) |  | 5.8 (4.3 – 7.8) |  | 5.6 (4.2 – 7.6) | |  | 2.3 (1.5 – 3.7) | |  |
| Child skin colour |  | 0.562 |  | 0.004 |  | | 0.020 |  | | 0.828 |
| White | 11.9 (10.1 – 13.9) |  | 3.8 (2.8 – 5.1) |  | 4.1 (3.1 – 5.4) | |  | 1.8 (1.2 – 2.7) | |  |
| Black | 14.1 (9.9 – 19.6) |  | 6.5 (3.8 – 10.9) |  | 8.9 (5.9 – 13.2) | |  | 2.4 (1.1 – 5.3) | |  |
| Brown | 11.1 (7.6 – 15.8) |  | 9.4 (6.2 – 13.8) |  | 5.3 (3.3 – 8.4) | |  | 2.2 (1.1 – 4.5) | |  |
| Other | 15.7 (8.9 – 26.2) |  | 4.3 (1.4 – 12.5) |  | 3.4 (1.1 – 10.1) | |  | 1.1 (0.2 – 7.6) | |  |
| Child hyperactivity |  | 1.000 |  | 0.553 |  | | 0.080 |  | | 0.087 |
| No | 12.1 (10.5 – 13.9) |  | 4.8 (3.8 – 6.0) |  | 4.5 (3.6 – 5.6) | |  | 1.8 (1.3 – 2.5) | |  |
| Yes | 11.7 (7.5 – 17.8) |  | 5.8 (3.1 – 10.9) |  | 8.1 (4.4 – 14.4) | |  | 4.0 (1.7 – 9.3) | |  |
| Child conduct problems |  | 0.054 |  | 0.024 |  | | 0.007 |  | | 0.147 |
| No | 11.0 (9.3 – 13.0) |  | 4.1 (3.1 – 5.5) |  | 4.0 (3.1 – 5.2) | |  | 1.7 (1.1 – 2.5) | |  |
| Yes | 14.7 (11.6 – 18.4) |  | 7.1 (5.0 – 10.0) |  | 7.5 (5.3 – 10.6) | |  | 2.9 (1.6 – 5.1) | |  |
| Home stimulation^a^ (tertiles) |  | 0.113 |  | 0.485 |  | | 0.505 |  | | 0.743 |
| 1^st^ (least stimulated) | 22.0 (14.3 – 32.2) |  | 8.5 (4.1 – 16.9) |  | 6.4 (2.9 – 13.5) | |  | 4.3 (1.6 – 10.8) | |  |
| 2^nd^ | 13.0 (6.9 – 23.3) |  | 5.8 (2.2 – 14.5) |  | 2.6 (0.7 – 9.9) | |  | 2.6 (6.5 – 9.9) | |  |
| 3^rd^ (most stimulated) | 9.7 (4.4 – 20.0) |  | 3.2 (0.8 – 12.1) |  | 3.4 (1.1 – 10.1) | |  | 2.3 (0.6 – 8.7) | |  |
| Child resting heart rate (tertiles) |  | 0.273 |  | 0.086 |  | | 0.409 |  | | 0.866 |
| 1^st^ (lowest) | 11.3 (9.1 – 14.0) |  | 6.1 (4.5 – 8.2) |  | 4.8 (3.2 – 7.0) | |  | 2.1 (1.2 – 3.8) | |  |
| 2^nd^ | 11.5 (9.0 – 14.6) |  | 3.3 (2.1 – 5.3) |  | 5.8 (4.2 – 7.9) | |  | 1.9 (1.1 – 3.4) | |  |
| 3^rd^ (highest) | 14.4 (11.3 – 18.2) |  | 5.1 (3.4 – 7.8) |  | 4.2 (2.9 – 6.0) | |  | 1.7 (1.0 – 3.0) | |  |
| Child IQ^a^ |  | 1.000 |  | 0.555 |  | | 0.745 |  | | 1.000 |
| <90 points | 16.2 (10.1 – 24.8) |  | 7.1 (3.4 – 14.2) |  | 3.1 (1.0 – 9.1) | |  | 3.1 (1.0 – 9.1) | |  |
| ≥90 points | 15.3 (9.7 – 23.3) |  | 4.5 (1.9 – 10.4) |  | 4.5 (2.2 – 9.1) | |  | 2.6 (1.0 – 6.7) | |  |
| Number of grade repetitions |  | 0.039 |  | 0.126 |  | | 0.002 |  | | 0.729 |
| 0 | 8.6 (6.2 – 11.8) |  | 2.8 (1.5 – 5.0) |  | 3.0 (2.0 – 4.6) | |  | 1.6 (0.9 – 2.9) | |  |
| 1 | 11.7 (8.5 – 16.0) |  | 5.2 (3.1 – 8.4) |  | 4.3 (2.6 – 6.9) | |  | 1.3 (0.6 – 3.2) | |  |
| 2 | 11.7 (8.8 – 15.4) |  | 5.2 (3.3 – 8.0) |  | 8.5 (6.0 – 11.9) | |  | 2.3 (1.1 – 4.5) | |  |
| 3 or more | 15.3 (12.0 – 19.4) |  | 6.2 (4.1 – 9.1) |  | 3.8 (2.0 – 7.2) | |  | 2.1 (0.9 – 5.0) | |  |
| School Completion |  | <0.001 |  | <0.001 |  | | 0.001 |  | | 0.048 |
| Did not finish school | 15.5 (13.2 – 18.2) |  | 8.1 (6.4 – 10.2) |  | 7.1 (5.4 – 9.3) | |  | 2.7 (1.7 – 4.2) | |  |
| Finished school | 8.9 (7.2 – 11.0) |  | 2.1 (1.3 – 3.3) |  | 3.6 (2.7 – 4.8) | |  | 1.5 (0.9 – 2.3) | |  |
| Total | **12.1 (10.6 – 13.8)** |  | **4.9 (4.0 – 5.9)** |  | **5.0 (4.0 – 6.1)** | |  | **1.9 (1.4 – 2.6)** | |  |

Note: 95%CI = 95% Confidence Interval

^a^measured at age 4 years for a sub-sample of the cohort; ^b^Fisher exact test
