# Supplementary material for: School Performance and Young Adult Crime in a Brazilian Birth Cohort
Source: J Dev Life Course Criminol. 2022 Oct 11;8(4):647–68. doi: 10.1007/s40865-022-00214-x (PMC9825356; doi:10.1007/s40865-022-00214-x)
Supplement: Supplementary file 4 — Supplementary file4 (DOCX 21 KB) [file 40865_2022_214_MOESM4_ESM.docx]

Article title: School performance and young adult crime in a Brazilian birth cohort
Journal name: Journal of Development and Life-course Criminology
Author names: [*hidden due to blindness*]
Affiliation: [*hidden due to blindness*]
E-mail address of the corresponding author: [*hidden due to blindness*]

**Supplementary Table 4.** Crude (n = 1,426 for grade repetitions and 1,662 for school completion) and adjusted (n = 1,352 for grade repetitions and 1,348 for school completion) associations between educational performance and crime among males in the 1993 Pelotas Birth Cohort Study

|  | MALE VIOLENT CRIME | | | | | |
| --- | --- | --- | --- | --- | --- | --- |
|  | Crude OR | 95%CI | Adjusted^a^ OR | 95%CI | Adjusted^a^ OR (IPW) | 95%CI (IPW) |
| Number of grade repetitions | *p* = 0.006^b^ | | *p* = 0.009^b^ | | *p* = 0.030^c^ | |
| 0 | 1.0 | Ref. | 1.0 | Ref. | 1.0 | Ref. |
| 1 | 1.4 | 0.9 – 2.3 | 1.3 | 0.8 – 2.2 | 1.3 | 0.7 – 2.1 |
| 2 | 1.4 | 0.9 – 2.3 | 1.4 | 0.8 – 2.3 | 1.4 | 0.8 – 2.3 |
| 3 or more | 1.9 | 1.2 – 3.0 | 1.9 | 1.2 – 3.2 | 1.9 | 1.2 – 3.2 |
| School Completion | *p*<0.001^c^ | | *p<*0.001^c^ | | *p* = 0.004^c^ | |
| Did not Finish School | Ref. | 1.00 | Ref. | 1.0 | 1.0 | Ref. |
| Finished School | 0.5 | 0.4 – 0.7 | 0.5 | 0.3 – 0.7 | 0.5 | 0.3 – 0.8 |
|  | MALE NON-VIOLENT CRIME | | | | | |
|  | Crude OR | 95%CI | Adjusted^a^ OR | 95%CI | Adjusted^a^ OR (IPW) | 95%CI (IPW) |
| Number of grade repetitions | *p* = 0.031^b^ | | *p* = 0.445^c^ | | *p* = 0.064^c^ | |
| 0 | 1.0 | Ref. | 1.0 | Ref. | 1.0 | Ref. |
| 1 | 1.9 | 0.9 – 4.2 | 1.6 | 0.7 - 3.8 | 1.6 | 0.7 – 3.8 |
| 2 | 1.9 | 0.9 – 4.1 | 1.7 | 0.7 – 3.8 | 1.7 | 0.7 – 4.3 |
| 3 or more | 2.3 | 1.1 – 4.8 | 1.9 | 0.8 – 4.3 | 2.0 | 0.8 – 4.9 |
| **School Completion** | p<0.001^c^ | | p<0.001^c^ | | *p* = 0.102^c^ | |
| Did not Finish School | Ref. | 1.00 | Ref. | 1.00 | 1.0 | Ref. |
| Finished School | 0.2 | 0.1 – 0.4 | 0.2 | 0.1 – 0.4 | 0.2 | 0.1 – 0.5 |

Notes: OR = Odds Ratio; CI = Confidence Interval; IPW = inverse probability weighting

^a^Adjusted for neighbourhood conditions, family income, maternal schooling, maternal belief in education, maternal mental health problems, harsh parenting, child skin colour, child hyperactivity, child conduct problems, child resting heart rate. For analyses of school completion, number of grade repetitions was also adjusted for.

^b^*p* value for trend

^c^*p* value for heterogeneity
