# Supplementary material for: School Performance and Young Adult Crime in a Brazilian Birth Cohort
Source: J Dev Life Course Criminol. 2022 Oct 11;8(4):647–68. doi: 10.1007/s40865-022-00214-x (PMC9825356; doi:10.1007/s40865-022-00214-x)
Supplement: Supplementary file 6 — Supplementary file6 (DOCX 18 KB) [file 40865_2022_214_MOESM6_ESM.docx]

Article title: School performance and young adult crime in a Brazilian birth cohort
Journal name: Journal of Development and Life-course Criminology
Author names: [*hidden due to blindness*]
Affiliation: [*hidden due to blindness*]
E-mail address of the corresponding author: [*hidden due to blindness*]

**Supplementary Table 6.** Overall crude (n = 3,079 for grade repetitions and n = 3,579 for school completion) and adjusted (n = 2,933 for grade repetitions and n = 2,929 for school completion) associations between school performance and crime and stratified by males (crude: n = 1,426 for grade repetitions and 1,662 for school completion; adjusted: n = 1,352) and females (crude: n = 1,653 for grade repetitions and n = 1,917 for school completion; adjusted n= 1,581) in the 1993 Pelotas Birth Cohort Study

|  | TOTAL SAMPLE – VIOLENT CRIME | | | | | | | | FEMALE VIOLENT CRIME | | | | MALE VIOLENT CRIME | | | | | |
| --- | --- | --- | --- | --- | --- | --- | --- | --- | --- | --- | --- | --- | --- | --- | --- | --- | --- | --- |
|  | Crude OR | | 95%CI | | Adjusted^a^ OR | | | 95%CI | Crude OR | 95%CI | Adjusted^a^ OR | 95%CI | Crude OR | 95%CI | Adjusted^a^ OR | | 95%CI | |
| Number of grade repetitions | *p*<0.001^b^ | | | | *p*<0.001^b^ | | | | *p* = 0.008^b^ | | *p* = 0.031^b^ | | *p* = 0.017^b^ | | *p* = 0.006^b^ | | | |
| 0 | 1.0 | Ref. | | | 1.0 | | Ref. | | 1.0 | Ref. | 1.0 | Ref. | 1.0 | Ref. | 1.0 | | Ref. | |
| 1 or more | 2.0 | 1.5 – 2.7 | | | 2.0 | | 1.4 – 2.8 | | 1.9 | 1.2 – 3.2 | 1.9 | 1.0 – 3.4 | 1.6 | 1.1 – 2.4 | 1.5 | | 1.0 – 2.4 | |
| School Completion | *p* <0.001^d^ | | | | *p* <0.001^d^ | | | | *p=*0.001^b^ | | *p*=0.260^b^ | | *-* | | *-* | | | |
| Did not Finish School | 1.0 | Ref. | | | 1.0 | | Ref. | | Ref. | 1.00 | Ref. | 1.00 | - | - | - | | - | |
| Finished School | 0.5 | 0.4 – 0.6 | | | 0.5 | | 0.4 – 0.7 | | 0.5 | 0.3 – 0.8 | 0.7 | 0.4 – 1.3 | - | - | - | | - | |
|  | TOTAL SAMPLE – NON-VIOLENT CRIME | | | | | | | | FEMALE NON-VIOLENT CRIME | | | | MALE NON-VIOLENT CRIME | | | | | |
|  | Crude OR | | | 95%CI | | Adjusted^a^ OR | | 95%CI | Crude OR | 95%CI | Adjusted^a^ OR | 95%CI | Crude OR | 95%CI | | Adjusted^a^ OR | | 95%CI |
| Number of grade repetitions | *p* = 0.006^b^ | | | | | *p* = 0.037^b^ | | | *p* = 0.663^b^ | | *p* = 0.771^b^ | | *p* = 0.021^b^ | | | *p* = 0.031^b^ | | |
| 0 | 1.0 | | | Ref. | | 1.0 | | Ref. | 1.0 | Ref. | 1.0 | Ref. | 1.0 | Ref. | | 1.0 | | Ref. |
| 1 or more | 1.9 | | | 1.2 – 3.1 | | 1.4 | | 0.8 – 2.5 | 1.2 | 0.6 – 2.5 | 1.1 | 0.5 – 2.6 | 2.1 | 1.1 – 4.0 | | 1.7 | | 0.8 – 3.5 |
| **School Completion** | *p* <0.001^d^ | | | | | *p* <0.001^d^ | | | *p =* 0.069^b^ | | *p =* 0.258^b^ | | *-* | | | *-* | | |
| Did not Finish School | 1.0 | | | Ref. | | 1.0 | | Ref. | Ref. | 1.00 | Ref. | 1.00 | - | - | | - | | - |
| Finished School | 0.3 | | | 0.2 – 0.4 | | 0.3 | | 0.2 – 0.5 | 0.5 | 0.3 – 1.0 | 0.6 | 0.2 – 1.5 | - | - | | - | | - |

Notes: OR = Odds Ratio; CI = Confidence Interval

^a^Adjusted for neighbourhood conditions, family income, maternal schooling, maternal belief in education, maternal mental health problems, harsh parenting, child skin colour, child hyperactivity, child conduct problems, child resting heart rate. For analyses of school completion, number of grade repetitions was also adjusted for.

^b^*p* value for heterogeneity

- Results showed in Table 3
