# Supplementary material for: School Performance and Young Adult Crime in a Brazilian Birth Cohort
Source: J Dev Life Course Criminol. 2022 Oct 11;8(4):647–68. doi: 10.1007/s40865-022-00214-x (PMC9825356; doi:10.1007/s40865-022-00214-x)
Supplement: Supplementary file 7 — Supplementary file7 (DOCX 16 KB) [file 40865_2022_214_MOESM7_ESM.docx]

Article title: School performance and young adult crime in a Brazilian birth cohort
Journal name: Journal of Development and Life-course Criminology
Author names: [*hidden due to blindness*]
Affiliation: [*hidden due to blindness*]
E-mail address of the corresponding author: [*hidden due to blindness*]

Table 5. Direct and indirect effects of crime and school failure mediated by problematic alcohol use, drug use, friends’ drug use and income in males

| Number of grade repetitions | Non-violent crime | | Violent crime | |
| --- | --- | --- | --- | --- |
|  | Direct | Indirect | Direct | Indirect |
|  | OR (95% CI) | OR (95% CI) | OR (95% CI) | OR (95% CI) |
|  | Problematic alcohol use | | | |
| 0 | Ref. | Ref. | Ref. | Ref. |
| 1 | 1.2 (0.6 - 2.5) | 1.1 (1.0 - 1.2) | 1.4 (0.8 - 2.4) | 1.0 (1.0 - 1.1) |
| 2 | 1.6 (0.8 - 3.2) | 1.0 (1.0 - 1.1) | 1.5 (0.9 - 2.6) | 1.0 (1.0 - 1.1) |
| 3 or more | 1.7 (0.8 - 3.5) | 1.1 (1.0 - 1.2) | 1.7 (1.0 - 3.0) | 1.2 (1.0 - 1.3) |
|  | **Drug use** | | | |
| 0 | Ref. | Ref. | Ref. | Ref. |
| 1 | 1.4 (0.6 - 3.4) | 1.0 (0.9 - 1.2) | 1.3 (0.8 - 2.1) | 1.0 (0.9 - 1.1) |
| 2 | 1.4 (0.6 - 3.5) | 1.1 (0.9 - 1.3) | 1.3 (0.8 - 2.1) | 1.1 (1.0 - 1.2) |
| 3 or more | 1.5 (0.7 - 3.5) | 1.1 (1.0 - 1.3) | 1.7 (1.0 - 2.8) | 1.1 (1.0 - 1.2) |
|  | **Friends’ drug use** | | | |
| 0 | Ref. | Ref. | Ref. | Ref. |
| 1 | 1.4 (0.6 - 3.6) | 1.0 (0.9 - 1.1) | 1.4 (0.6 - 3.6) | 1.0 (0.9 - 1.1) |
| 2 | 1.4 (0.6 - 3.6) | 1.0 (0.9 - 1.2) | 1.4 (0.6 - 3.6) | 1.0 (0.9 - 1.2) |
| 3 or more | 1.4 (0.5 - 3.6) | 1.2 (1.1 - 1.3) | 1.4 (0.5 - 3.6) | 1.2 (1.1 - 1.3) |
|  | **Income level** | | | |
| 0 | Ref. | Ref. | Ref. | Ref. |
| 1 | 1.1 (0.3 - 4.0) | 1.1 (1.0 - 1.3) | 1.3 (0.7 - 2.5) | 1.0 (1.0 - 1.1) |
| 2 | 1.3 (0.4 - 4.9) | 1.1 (1.0 - 1.3) | 1.3 (0.7 - 2.5) | 1.0 (1.0 - 1.1) |
| 3 or more | 1.4 (0.4 - 4.8) | 1.1 (1.0 - 1.4) | 1.7 (0.9 - 3.2) | 1.0 (1.0 - 1.1) |
