# Supplementary figures and images for: School Performance and Young Adult Crime in a Brazilian Birth Cohort
Source: J Dev Life Course Criminol. 2022 Oct 11;8(4):647–68. doi: 10.1007/s40865-022-00214-x (PMC9825356; doi:10.1007/s40865-022-00214-x)

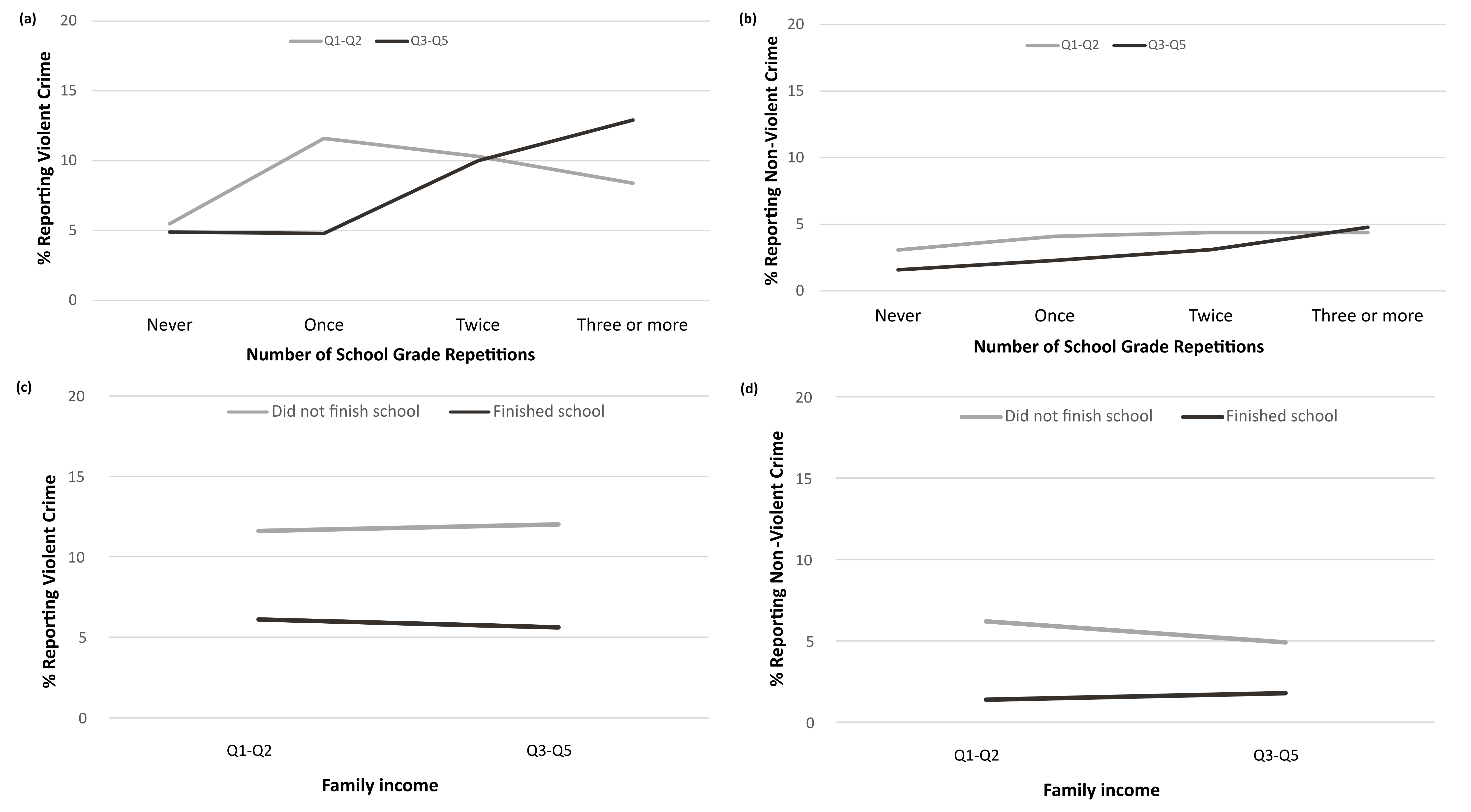

Supplement: Supplementary file 8 — Supplementary file8 (PNG 502 KB) [file 40865_2022_214_MOESM8_ESM.png]
